# P02.18. Clinically meaningful differences in pain and disability after cupping treatment for chronic neck pain: reanalysis of 4 randomized controlled trials

**DOI:** 10.1186/1472-6882-12-S1-P74

**Published:** 2012-06-12

**Authors:** R Lauche, G Dobos, H Cramer

**Affiliations:** 1 University of Duisburg, Essen, Germany

## Purpose

The assessment of clinically meaningful differences in patients’ self-reported outcomes has become more important when interpreting the results of clinical studies. In conventional medicine these assessments are common; however, there is still doubt whether these results apply to complementary trials. The aim of this analysis is the determination of minimal clinically important differences (MCID) and substantial clinical benefits (SCB) in patients with chronic non-specific neck pain after cupping treatment. The MCID and SCB for pain intensity (VAS) and neck disability index (NDI) were estimated using common anchor-based methods.

## Methods

The data set comprises a total of 200 patients participating in clinical trials on cupping therapy. The MCID and SCB for VAS and NDI were determined using receiver operating characteristic (ROC) curve analysis with an adapted assessment of change in health status (SF36) as an anchor. This item asks the patients how they would rate their health in general now, compared to before treatment. Answer categories ranged from "much better", "somewhat better", "about the same", "somewhat worse" to “much worse." An ROC curve was constructed for each outcome. MCID derived from the ROC was the score with equal sensitivity and specificity to distinguish "somewhat better" from "about the same". The SCB was the score to distinguish "much better" from "somewhat better".

## Results

The calculated MCID was 8mm/21% for VAS and 3points/10.2% for NDI. The SCB was 26.5mm/66.1% for VAS and 8.4points/29.7% for NDI.

## Conclusion

MCID and SCB for VAS in cupping trials are comparable to conventional trials although absolute and relative changes were slightly different. For NDI absolute and relative changes are smaller than in other trials, which might be due to the fact that scores were low in general. Altogether results support the assumption that patients’ perceptions of treatment benefits in complementary trials are largely comparable to those in conventional trials.

